# Research on the Interaction Mechanism Between α Mino-Phosphonate Derivative Q-R and Harpin-Binding Protein 1 in Tobacco (*Nicotiana tabacum*) Plants

**DOI:** 10.3389/fmicb.2021.621875

**Published:** 2021-03-23

**Authors:** Maoxi Huang, Yunlong Yan, Li Wang, Jun Chen, Tao Liu, Xin Xie, Xiangyang Li

**Affiliations:** ^1^State Key Laboratory Breeding Base of Green Pesticide and Agricultural Bioengineering, Key Laboratory of Green Pesticide and Agricultural Bioengineering, Ministry of Education, Guizhou University, Guiyang, China; ^2^College of Agriculture, Guizhou University, Guiyang, China

**Keywords:** Q-R, HrBP1, PR-1a, interaction, binding

## Abstract

Amino-phosphonate derivative R-diphenyl-1-(4-methylbenzothiazole-2-amino)-1-(thiphene-2-yl)-methylphosphonate (Q-R) has a high protective anti-tobacco mosaic virus (TMV) activity. However, the mechanism responsible for Q-R’s effect on TMV infection is largely unknown. Here, we studied the expression levels of harpin-binding protein 1 (HrBP1) and pathogenesis-related protein-1a (PR-1a) in TMV-infected tobacco plants by using reverse transcription quantitative real-time PCR. Then, we verified the interactions between Q-R and the HrBP1 protein from *Escherichia coli* using isothermal titration calorimetry and studied the Q-R-associated assembly of HrBP1 using size-exclusion chromatography. The results showed that the expression levels of HrBP1 and PR-1a genes were significantly increased by Q-R at the transcriptional level in TMV-infected tobacco plants, and the *E. coli*-expressed HrBP1 protein was assembled into oligomers by Q-R via binding to HrBP1 with a dissociation constant of 1.19 μM. We, therefore, concluded that Q-R activated the HrBP1 and PR-1a genes and enhanced the ability of HrBP1 to assemble in tobacco plants.

## Introduction

Harpin induces hypersensitive responses to plants and induces the defensive processes of pathogen-associated immunity in plant roots (El-Maarouf et al., 2001; Aljaafri et al., 2017; Lawaju et al., 2018).

Harpin-binding protein (HrBP1) is a protein receptor of Harpin. HrBP1 was first discovered in planting cell walls. HrBP1 induces systemic acquired resistance to plants (El-Maarouf et al., 2001). HrBP1 can be selectively up-regulate several signaling pathways, including those of salicylic acid (SA), ethylene and the jasmonic acid. In the SA pathway, the genes encoding enhanced disease susceptibility 1, non-expressor of pathogenesis- related 1, pathogenesis-related protein-1a (PR-1a), pathogenesis related- protein-2, pathogenesis-related protein-5, and alternative oxidase were activated. In the ethylene and jasmonic acid pathways, PDF1.2 and Thi2.1 were activated. In addition, HrBP1 generates disease resistance by up-regulating the harpin-induced gene related to the *mitogen-activated protein kinase* gene (Lee et al., 2001; Wang et al., 2002; Liu et al., 2006; Penmetsa et al., 2008; Camehl et al., 2010). HrBP1 expression results in the hypersensitive reaction and systemic acquired resistance related to the *non-race-specific disease resistance* and *enhanced disease susceptibility-1* genes (Peng et al., 2003; Gopalan, 2008). Thus, HrBP1 can generate anti-viral responses in plants.

**FIGURE 1:**
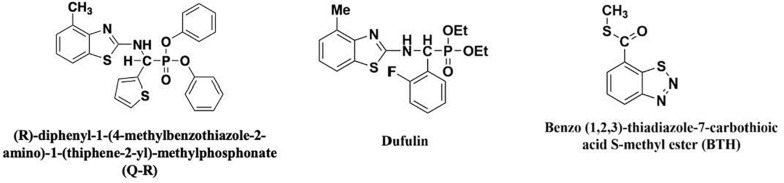
The chemical structures of antiviral agents.

In a previous study, we found that dufulin-associated activation of HrBP1 leads to the SA signaling pathway and produces antiviral responses from the tobacco plants (Chen et al., 2012). Amino-phosphonate derivative (R)-diphenyl-1-(4-methylbenzothiazole-2-amino)-1-(thiphene-2-yl)-methylphosphonate (Q-R) is a derivative compound of dufulin with high bioactivities on tobacco mosaic virus (TMV) and cucumber mosaic virus (Song et al., 2009; Zhang et al., 2016, 2017). However, the mechanism of Q-R that affects TMV infection is largely unknown. The structure of Q-R is similar to that of dufulin (Figure 1). To further to investigate the protective mechanism of Q-R, we studied the expression levels of the *HrBP1* and *PR-1a* genes in TMV-infected tobacco (*Nicotiana tabacum*) plant treated with Q-R. Then, we studied the mechanism of Q-R affecting by HrBP1 protein.

## Materials and Methods

### Antiviral Agents and Plant Materials

The chiral α-aminophosphonate derivative Q-R (99%) and dufulin (99%) was synthesized in our laboratory (Zhang et al., 2016). Benzo-1, 2, 3-thiadiazole-7-carbothioic acid S-methyl ester (BTH) (99%) was purchased from Aladdin Biochemical Technology Co. (Shanghai, China) (Sleiman et al., 2017). The structural formulae of these compounds are presented in Figure 1. In total, ∼30 *Nicotiana tabacum* K326 seeds were placed in a growth chamber at 25°C, after 12 weeks, the tobacco plants were used for the study. The TMV was purified as previously described by Gooding (Gooding and Hebert, 1967). The 12-weeks-old tobacco plants were sprayed with 5 mL of 500-μg/mL Q-R three times for 1-d intervals (500-μg/mL dufulin and BTH were positive control). Then, the plants were inoculated with TMV and grown for 5 days. The leaf samples of tobacco plants were harvested for further analyses. Three independent experimental replicates were conducted. The control tobacco plants, not exposed to TMV, were kept in another growth chamber.

### Relative Expression Levels of Activated Response Genes After TMV-Infected Tobacco Were Treated With Antiviral Agents as Assessed by Reverse Transcription Quantitative Real-Time PCR (qPCR)

To verify the effects of Q-R on the expression of antiviral response genes in the TMV-infected tobacco plants, the expression levels of the *HrBP1* and *PR-1a* genes were examined using qPCR. Briefly, 100 mg total RNA from tobacco samples (treated with 500 μg/mL of an antiviral agent) was extracted using the TRIzol reagent kit, independently (TakaRa, Japan), and cDNAs were synthesized using a Reverse Transcription System kit (TakaRa). Then, qPCR was performed using SYBR Premix Ex TaqII (TaKaRa). Gene expression levels were normalized using β*-actin* as the internal control (Supplementary Table 1; Chen et al., 2012; Gao et al., 2019). The relative expression gene levels were calculated using the 2^–ΔΔCt^ method (Livak and Schmittgen, 2001; Wang et al., 2019).

### HrBP1 Plasmid Constructs

The sequence (GenBank accession no. 107762961) for *N. tabacum HrBP1* was used to design a forward primer containing a *Nde*I (underlined) restriction site in (5′-GGAAT TCCATATGGCTTCTCTACTTCAGTACTCTACACT-3′) and a *Xho*I (underlined) restriction site in reverse primers (5′- CCGC TCGAGTGAGATAACGAAAACTCTAAGCTCTC-3′). The full- length *N. tabacum HrBP1* gene (831 bp) was inserted into pET28a (Novagen, United States), resulting in the plasmid pET28a-HrBP1. The *N. tabacum HrBP1* gene was confirmed by 1% agarose gel electrophoresis and the *N. tabacum HrBP1* DNA sequence was confirmed at the Shanghai Sangon Company (Chen et al., 2012).

### HrBP1 Expression and Purification

The plasmid pET28a-HrBP1 in a culture of *E. coli* strains BL21(DE3) (Novagen) was transferred to 1 L of Luria broth (30-μg/mL kanamycin). When the OD_600_ of Luria broth reached 0.65, the temperature was decreased to 16°C, and 0.8–1.0 mM isopropyl-β-D-galactopyranoside (IPTG) was supplemented in Luria broth. HrBP1 protein was expressed in Luria broth overnight. Then, the cells containing HrBP1 protein were collected, harvested, and resuspended in 50 mM Tris-HCl, 150 mM NaCl and 1 mM β-mercaptoethanol buffer (pH 7.5) and the cells were lysed at 4°C using sonication (Li et al., 2013, 2015a). The whole cells were centrifuged at 12,000 g for 30 min at 4°C, the soluble supernatants were collected and loaded onto a 5-mL HisTrap high-performance column attached to an AKTA purifier protein liquid chromatography system (GE Healthcare, United States), and the protein was eluted by 20 mM Tris-HCl and 300-mM imidazole buffer (pH 7.5). Eluate fractions were collected and analyzed by 12% sodium dodecyl sulfate (SDS)-polyacrylamide gel electrophoresis (PAGE), and the crude protein were extracted at 4°C using a desalting column (GE Healthcare), and the eluate fractions were pooled and concentrated by ultrafiltration (10-kDa cut-off). The protein amount was detected by absorbance at 280 nm using a Genequant100 (GE Healthcare).

### Size-Exclusion Chromatography (SEC), PAGE and High-Resolution Mass Spectrometry

At 4°C, SEC was performed using Superdex 200 10/300 GL column (GE Healthcare) attached to an AKTA purifier protein liquid chromatography system (GE Healthcare) (Li et al., 2015b). The column was equilibrated using a buffer containing 20 mM Tris-HCl and 100 mM NaCl (pH 7.5). Myoglobin (17 kDa), ovalbumin (43 kDa), albumin (67 kDa), IgG (158 kDa), and ferritin (440 kDa) was used as the molecular mass standards (Bio-Rad, United States). The protein content was detected at a wavelength of 280 nm. The purified protein was confirmed by 8% enriching gel and 12% SDS-PAGE, and then stained with Coomassie brilliant blue (Li et al., 2015a).

The purified HrBP1 proteins were digested using trypsin, the digested products were analyzed using a Nano LC-1DTM plus system (Eksigent Technologies, United States) and TripleTOF 5600 MS (AB SCIEX, United States) (Gopalan, 2008; MacLean et al., 2010; Chen et al., 2015; Yang et al., 2017; Shi et al., 2018; Yu et al., 2018). The achieved peptide masses were searched and blasted using UniProt database without any species or peptide limits. Multiple peptide fragments matched the reported HrBP1 segments (Uniprot accession no.: Q5QJB2). High-resolution masses spectrometry data were extracted and retrieved using PeakView (AB SCIEX) and ProteinPilot (AB SCIEX). The trypsin cleaved sites and the predicted m/z values were analyzed using Skyline (AB SCIEX) (Huntley et al., 2014).

### Isothermal Titration Calorimetry (ITC)

At 25°C, ITC-based binding experiments were performed using an ITC 200 Micro Calorimeter (GE Healthcare). The HrBP1 protein was in the buffer containing 20-mM Tris-HCl and 100-mM sodium chloride (pH 7.5). The 2 mM Q-R compound was titrated into the HrBP1 protein (0.1 mM) in a 200-μL sample cell as follows: 0.4 μL for the first injection and 2 μL for the next 19 injections using a 40-μL microsyringe, the intervals of each injection was150 s. According to the manufacturer’s instructions, the integrated heat data were analyzed using the one-set-of-sites model. The first data point was not required in the analysis. The binding parameters reaction including enthalpy change, ΔH (cal⋅mol^–1^), binding constant K (mol^–1^) and number of molecules per HrBP1 protein (n) was floating in the fit. The binding free energy and reaction entropy was calculated (Kumar et al., 2014). The dissociation constant K_*d*_ was determined as 1/K (Kumar et al., 2014).

### Statistical Analyses

Data were submitted to an analysis of variance (ANOVA) using Duncan’s multiple range test by SPSS 19.0 software (IBM, United States). *P* < 0.05 was identified as the significance level.

## Results and Discussion

### Relative Expression Levels of Antiviral Response Genes After Q-R Treatment in TMV-Infected Tobacco as Assessed by qPCR

**FIGURE 2:**
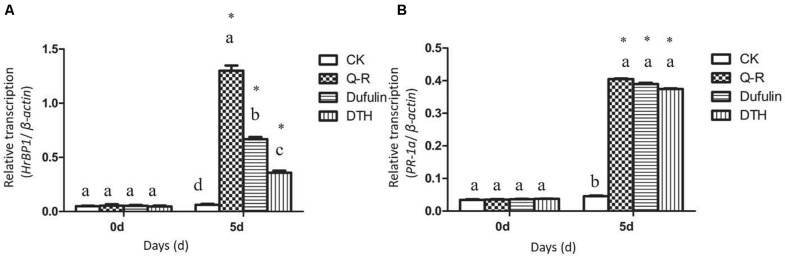
Antiviral response genes expression analysis by qPCR using gene-specific primers (Supplementary Table 1). Relative expression of the *HrBP1* **(A)** and *PR-1a* **(B)** genes in TMV-infection plants treated with Q-R, dufulin and BTH at 500 μg/mL at 5 days. Asterisks represent the significantly different between agent treatment and control (*P* < 0.05). The different lowercase letters represent *HrBP1*/*PR-1a* genes expression values in the different treatment groups (*P* < 0.05).

To investigate whether Q-R inhibits the antiviral response genes’ expression levels in TMV-infected tobacco plants, we detected the relative expression of *HrBP1* and *PR-1a* utilizing the qPCR assay. The expression levels of the *HrBP1* and *PR-1a* genes in TMV-infected tobacco plants significantly increased compared with the CK (*P* < 0.05) at the transcriptional level after exposure to by 500 μg/mL of Q-R on 5 days (Figure 2). Compared with dufulin and BTH, the expression levels of the *HrBP1* genes were significantly (*P* < 0.05) promoted by compound Q-R (Figure 2A).

### Cloning of the HrBP1 Gene

**FIGURE 3:**
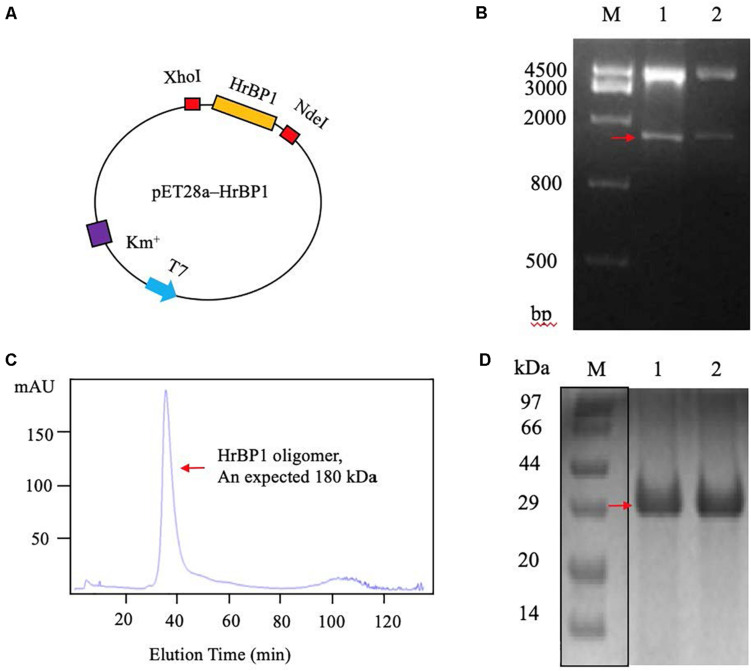
The *HrBP1* gene determination and HrBP1 protein expression and purification. **(A)** Recombinant vector pET28a-HrBP1 was digested by *Nde*I and *Xho*I. **(B)** The *HrBP1* gene size (ca. 831 bp) was confirmed by 1% agarose gel electrophoresis. Lane M is a DNA marker; Lanes 1 and 2 shows the digestion of *HrBP1* gene by *Nde*I and *Xho*I in the pET28a-HrBP1 recombinant plasmid; the red arrow shows bands of HrBP1 bands. **(C)** HrBP1 oligomer protein with expected size 180 kDa between 440 kDa (Ferritin) and 158 kDa (IgG). **(D)** M is protein Marker; Lanes 1 and 2 are HrBP1 purification proteins in 12% SDS-PAGE; the red arrow shows HrBP1 bands.

To obtain the code sequence of the HrBP1 gene in tobacco plant, we amplified the full-length cDNA sequence of HrBP1 and cloned it into the pET28a plasmid (Figure 3A). Electrophoresis and furthering sequence of the fragment confirmed the HrBP1 gene having an expected 831-bp size and nucleotide sequence (Figure 3B and Supplementary Figure 1) as reported (Chen et al., 2012).

### Expression and Purification of HrBP1 Using the pET28a Vector

To obtain sufficient quantities of HrBP1 protein for further analyses, we designed the pET28a expression vector including the HrBP1 gene and transformed it into *E. coli* strain BL21(DE3). The expressed protein was supported by SDS-PAGE analysis to be 30 kDa. The HrBP1 protein was overexpressed after exposure to 0.8 mM isopropyl-β-D-galactopyranoside (IPTG) at 16°C for 16 h (Figures 3C,D). The expressed HrBP1 proteins (approximately 90%) was purified and concentrated on subsequent analysis.

### Identification and Analysis of the Expressed HrBP1 Protein

To further determine the amino acid composition of the expressed HrBP1 protein through a polypeptide analysis. The 18.1% fraction of the obtained polypeptide fragments covered the entire 276 amino acids, and the sequence was equally expected.

The SEC results showed that the HrBP1 recombinant protein was an oligomer. The HrBP1 protein was elected and calculated to be approximately 180 kDa based on a reference size (Figure 3C). The SDS-PAGE analysis results showed that the size of the collected protein was approximately 30 kDa (Figure 3D), which indicated that the HrBP1 oligomer was destroyed by SDS *in vitro*.

### Interactions Between Q-R and the HrBP1 Protein

**FIGURE 4:**
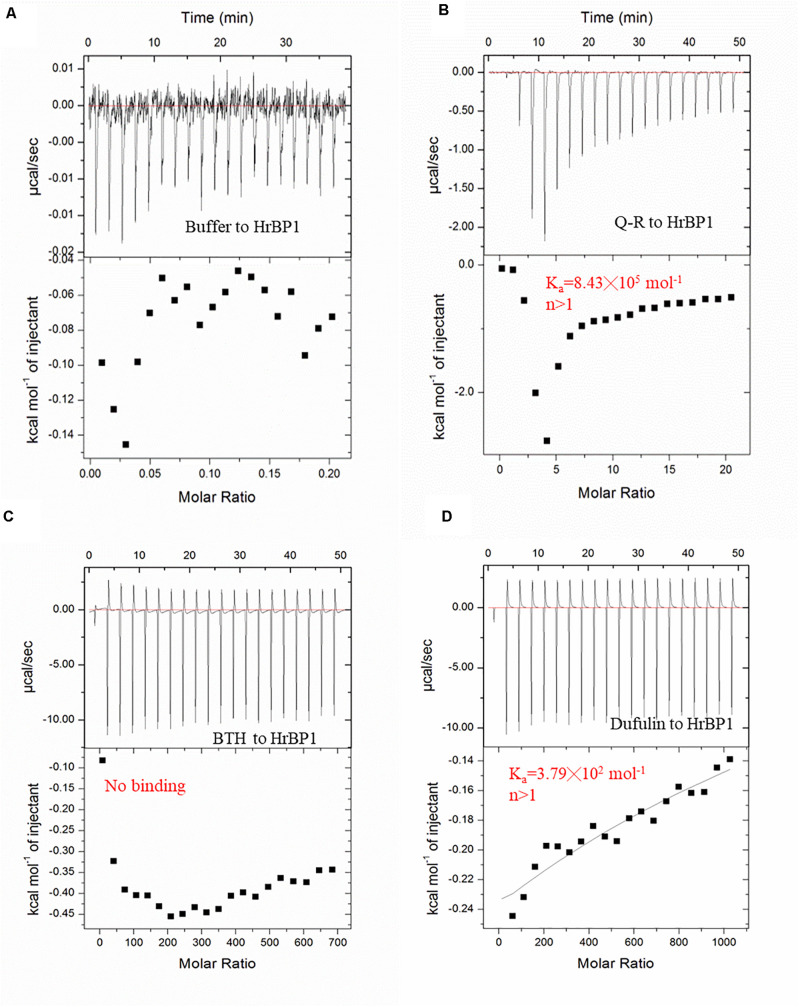
ITC studies of Q-R, dufulin, BTH and HrBP1 purification protein. **(A)** control. **(B)** Q-R and HrBP1. **(C)** BTH and HrBP1. **(D)** Dufulin and HrBP1.

To examine the interaction between Q-R and the HrBP1 protein, we analyzed the binding affinity between Q-R to HrBP1 protein using ITC. The binding affinity with Q-R to the HrBP1 protein was stronger than that of the control of antiviral agents BTH and dufulin. Q-R on the HrBP1 protein had a Kd value of 1.19 μM (8.43 × 10^5^ mol^–1^, Figures 4A,B). The titration data indicated an apparent negative enthalpy values (ΔG ≈−8.08) during the binding of Q-R to the HrBP1 protein. While no binding occurred between the BTH and HrBP1 protein (Figures 4A,C), and weak binding occurred between the dufulin and HrBP1 protein (Figures 4A,D). Thus, the HrBP1–Q-R complex was stable, implying that Q-R had the ability to bind the HrBP1 protein.

### Confirmation of the Protein-Ligand Interactions Between Q-R and HrBP1

**FIGURE 5:**
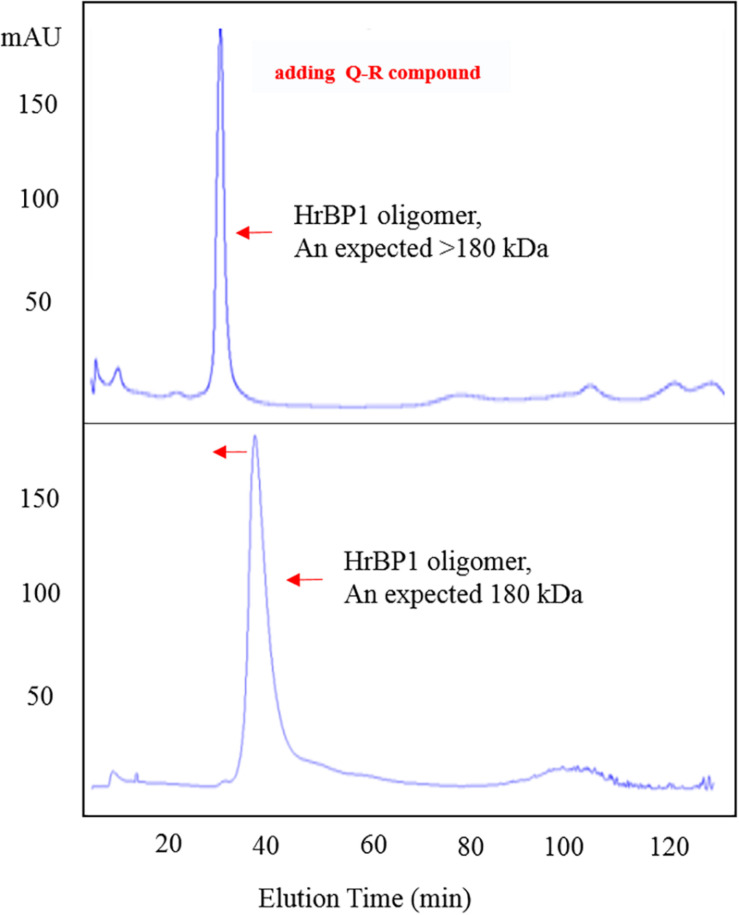
Q-R induced the assembly of HrBP1 large oligomer protein with expected size > 180 kDa between 440 kDa (Ferritin) and 158 kDa (IgG).

**FIGURE 6:**
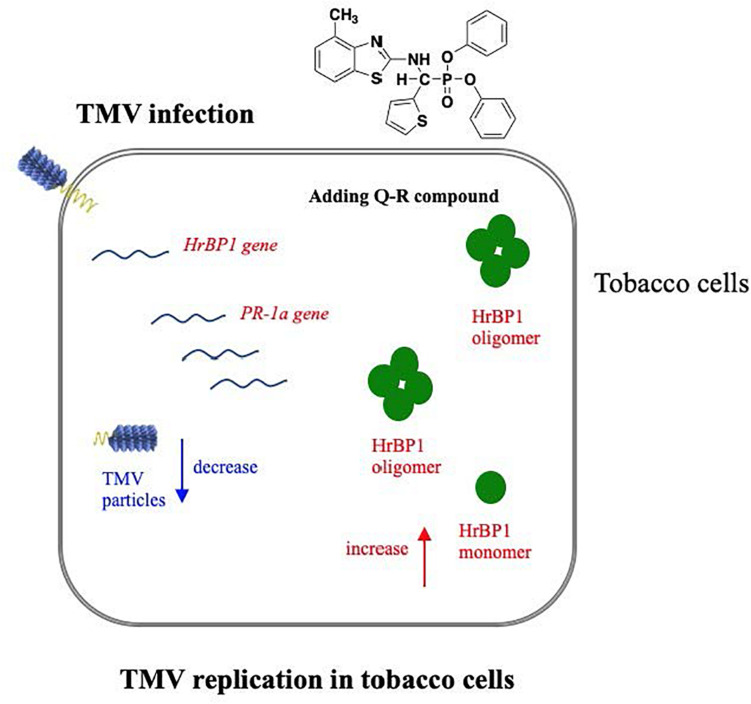
Hypothetical pathway wherein the HrBP1 protein of tobacco plant was induced by Q-R through promotes the expression of the *HrBP1* and *PR-1a* genes in tobacco plants, which leads to Q-R induces antiviral responses and inhibits the duplication of TMV in TMV-infected tobacco plant. Red indicate the up-regulated genes/protein, blue indicate the down-regulated genes/protein.

To confirm the interaction between Q-R and the HrBP1 protein, we analyzed the HrBP1 protein’s state of aggregation after being treated by Q-R using the SEC. Q-R induced the assembly of a large HrBP1 protein oligomer. Briefly, the HrBP1 protein formed functional aggregates. After adding Q-R, the protein samples were examined by SEC. We further assessed the effects of Q-R on the biochemical properties of the HrBP1 protein. As expecting, the HrBP1 protein was present in a large aggregated state (Figure 5). Thus, we deduced that Q-R induces the assembly of the HrBP1 protein and the further formation of the HrBP1–Q-R complex (Figure 6), which agreed with the ITC studies.

## Conclusion

In summary, our study clarified the mechanism by which Q-R produces antiviral responses associated with the HrBP1 protein expressed in *E. coli* (*In vitro*) and tobacco plants (*In vivo*). *In vitro*, a strong affinity existed on Q-R and the HrBP1 protein. *In vivo*, Q-R promoted the expression of the HrBP1 gene in tobacco plants. Thus, Q-R produces antiviral responses by activating the HrBP1 gene and protein in tobacco plants.

## Data Availability Statement

The data presented in the study are deposited in the NCBI repository, accession number MW256718.

## Author Contributions

XL designed this study. MH carried out the clone and protein expression studies, participated in the sequence alignment, and drafted the manuscript. YY, LW, JC, and TL performed MST and qPCR tests. XX and XL critically revised the manuscript. All authors read and approved the final version of the manuscript.

## Conflict of Interest

The authors declare that the research was conducted in the absence of any commercial or financial relationships that could be construed as a potential conflict of interest.

## Supplementary Material

Supplementary Figure 1 Nucleotide sequence and amino acid sequence of *HrBP1* sequence from *Nicotiana tabacum*.

Supplementary Table 1 Primer sequences used in qPCR.

## Abbreviations

Q-R: R-diphenyl -1-(4-methylbenzothiazole-2-amino)-1-(thiphene-2-yl)-methylphosphonate
TMV: tobacco mosaic virus
HrBP1: harpin-binding protein 1
PR-1a: pathogenesis-related protein-1a
BTH: Benzo-1, 2, 3-thiadiazole-7-carbothioic acid S-methyl ester
SA: salicylic acid
IPTG: isopropyl- β -D-galactopyranoside
SEC: Size-exclusion chromatography
SDS-PAGE: sodium dodecyl sulfate-polyacrylamide gel electrophoresis.
