# Supplementary material for: Research on the Interaction Mechanism Between α Mino-Phosphonate Derivative Q-R and Harpin-Binding Protein 1 in Tobacco (Nicotiana tabacum) Plants
Source: Front Microbiol. 2021 Mar 23;12:621875. doi: 10.3389/fmicb.2021.621875 (PMC8044911; doi:10.3389/fmicb.2021.621875)
Supplement: Supplementary Figure 1 — Nucleotide sequence and amino acid sequence of HrBP1 sequence from Nicotiana tabacum. [file Data_Sheet_1.PDF]

Supplementary materials

Research on the interaction mechanism between α mino-phosphonate derivative Q-R and harpin-binding protein 1 in tobacco (*Nicotiana tabacum*) plants

Maoxi Huang[a1], Yunlong Yan[a,b1], Li Wang[a,b1], Jun Chen[a,b], Tao Liu[a], Xin Xie[b], Xiangyang Li*[a]

[a]State Key Laboratory Breeding Base of Green Pesticide and Agricultural Bioengineering, Key Laboratory of Green Pesticide and Agricultural Bioengineering, Ministry of Education, Guizhou University, Guiyang 550025, PR China

[b]College of Agriculture, Guizhou University, Guiyang 550025, PR China

[1]these authors contributed equally to this work

*** Correspondence:**

Xiangyang Li

xyli1@gzu.edu.cn

Supplementary Figure 1 Nucleotide sequence and amino acid sequence of HrBP1 sequence from *Nicotiana tabacum*.

Harpin binding protein sequence from *Nicotiana tabacum*

Nucleotide sequence (831 bp)

```
ATGGCCTCCCTGCTGCAATATTCCACTCTGCCGCTGTCTAACAATCATTGCAGCTCCTCTCTGCCGAGCCTGACGTG
TCACCTGAGCAAACGTTCCAACCGCAACACCCAGAAACTGCTGGAGAAGAAGAAATACCACATCAAAAAGTCTC
TGATCTGCCAGAGCGGTATCGACGAACTGGCATTCATCGAACTGCCGGGCACGAAAGAAGCTAAAGCAGAACTGA
TCGGCTCCCTGAAACTGAAGCTGCTGTCTGCGGTAAGCGGTCTGAACCGCGGTCTGGCGGCATCCGAAGAAGACC
TGAAAAAAGCCGATGCGGCTGCTAAAGAGCTGGAATCTTGTGCAGGCGCTGTTGATCTGAGCGCGGACCTGGATA
AACTGCAGGGCCGTTGGAAACTGATTTACTCTTCCGCTTTCTCTGGCCGTACTCTGGGTGGTTCTCGTCCGGGTCC
GCCAACCGGTCGTCTGCTGCCAATCACTCTGGGCCAGGTTTTCCAGCGCATCGACGTGCTGTCTAAAGACTTTGAT
AACATCGTAGAACTGGAACTGGGTGCACCTTGGCCGCTGCCTCCGGCCGAACTGACCGCGACCCTGGCGCACAAA
TTTGAGCTGATCGGCAGCTCTACCATCAAAATTACCTTCGAAAAAACCACTGTGAAAACTACTGGCATCCTGAGCC
AGCTGCCGCCATTCGAAGTTCCGCGTATTCCGGACCAACTGCGTCCGCCGTCCAACACCGGCTCCGGTGAATTTGA
AGTCACCTACATTGACTCTGATACCCGTGTGACTCGTGGTGATCGCGGTGAGCTGCGCGTTTTCGTTATTTCTTAA
```

Amino acid sequence (276 aa)

```
MASLLQYSTLPLSNNHCSSSLPSLTCHLSKRSNRNTQKLLEKKKYHIKKSLICQSGIDELAFIELPGTKEAKAELIGSLKLKLLSAVSGLNRGLAASEEDLKK
ADAAAKELESCAGAVDLSADLDKLQGRWKLIYSSAFSGRTLGGSRPGPPTGRLLPITLGQVFQRIDVLSKDFDNIVELELGAPWPLPPAELTATLAHKFELIG
SSTIKITFEKTTVKTTGILSQLPPFEVPRIPDQLRPPSNTGSGEFEVTYIDSDTRVTRGDRGELRVFVIS*
```

Supplementary Table 1 Primer sequences used in qPCR.

Gene name	Forward primers	Reverse primers
HrBP1	5'-gcttctctacttcagtactctacact-3'	5'-gataacgaaaactctaagctctc-3'
NPR1	5'-tagcgtattgcgatgcaaag-3'	5'-ttccatcggatgtcagatca-3'
β-actin	5'-ttgtccgtgacatgaaggag-3'	5'-atcatggatggctggaagag-3'
